# Defining Collective Priorities: Research and Learning Agendas for Family Planning Across 6 Countries

**DOI:** 10.9745/GHSP-D-22-00469

**Published:** 2023-08-28

**Authors:** Sarah Brittingham, Trinity Zan, Kouakou Hyacinthe Andoh, Kabita Aryal, Marcos Chissano, Olivia Ferguson, Jean Christophe Fotso, Issoufa Harou, Sangita Khatri, Kadidiatou Raïssa Kourouma, Suzanne N. Kiwanuka, Bibek Kumar Lal, Alda Mahumana Govo, Morrisa Malkin, Philip Mkandawire, Mary Mulombe Phiri, Charles Olaro, Ndola Prata, Shannon Pryor, Bhagawan Shrestha, Basant Thapa, Fatoumata Traoré Touré

**Affiliations:** a FHI 360, Durham, NC, USA.; b Programme National de Santé de la Mère et de l’Enfant, Ministère de la Sante, de l’Hygiène Publique et de la Couverture Maladie Universelle, Abidjan, Côte d’Ivoire.; c Family Welfare Division, Kathmandu, Nepal.; d Population Services International, Maputo, Mozambique.; e FHI360, Durham, NC, USA; Formerly of Population Services International, Washington, DC, USA.; f EVIHDAF, Yaoundé, Cameroon.; g Direction Générale de la Population et Santé de la Reproduction; Ministère de la Santé Publique, de la Population et des Affaires Sociales, Niamey, Niger.; h Save the Children International, Kathmandu, Nepal.; i Cellule de Recherche en Santé de la Reproduction de Côte d'Ivoire and EVIHDAF, Abidjan, Côte d’Ivoire.; j Makerere University College of Health Sciences, School of Public Health, Kampala, Uganda.; k Direcção Nacional de Saúde Pública, Ministerio da Saúde, Maputo, Mozambique.; l Population Services International, Lilongwe, Malawi.; m Reproductive Health Directorate, Ministry of Health, Lilongwe, Malawi.; n Curative Services, Ministry of Health, Kampala, Uganda.; o EVIHDAF and University of California at Berkeley, Berkeley, CA, USA.; p Save the Children US, Washington, DC, USA.; q FHI 360, Kathmandu, Nepal.; r FHI 360, Abidjan, Côte d’Ivoire.

## Abstract

Evidence should be the foundation for a well-designed family planning (FP) program, but existing evidence is rarely aligned with and/or synthesized to speak directly to FP programmatic needs. Based on our experience cocreating FP research and learning agendas (FP RLAs) in Côte d’Ivoire, Malawi, Mozambique, Nepal, Niger, and Uganda, we argue that FP RLAs can drive the production of coordinated research that aligns with national priorities.

To cocreate FP RLAs, stakeholders across 6 countries conducted desk reviews of 349 documents and 106 key informant interviews, organized consultation meetings in each country to prioritize evidence gaps and generate research and learning questions, and, ultimately, formed 6 FP RLAs comprising 190 unique questions. We outline the process for consensus-driven development of FP RLAs and communicate the results of an analysis of the questions in each FP RLA across 4 technical areas: self-care, equity, high impact practices, and youth. Each question was categorized as a learning versus research question, the former indicating an opportunity to synthesize existing evidence and the latter to conduct new research to answer the question. Themes emerging from the data shed light on shared evidence gaps across the 6 countries. We argue that similarities and differences in the questions in each FP RLA reflect the unique implementation experience and context, as well as each country’s placement on the FP S-curve. Early uses of the FP RLAs include informing the development of FP costed implementation plans and FP2030 commitments. FP RLAs have also been discussed in multiple thematic working groups. For FP stakeholders, these FP RLAs represent a consensus-based agenda that can guide the generation and synthesis of evidence to answer each country’s most pressing questions, ultimately driving progress toward increasingly evidence-based programming and policy.

## Abstract (precis)

Policymakers and national stakeholders can cocreate FP research and learning agendas to identify and prioritize evidence gaps and foster responsive research, thereby driving progress toward increasingly evidence-based FP programming and policy.

## Teaser

**Key Findings:**

- Family planning (FP) research and learning agendas (RLAs) build a comprehensive understanding of the FP research landscape, identify key evidence gaps, and define national priority questions to drive the production of knowledge that ushers progress toward FP goals.
- Despite having unique FP contexts, the 6 country FP RLAs share themes within self-care, equity, high impact practices, and adolescent and youth sexual and reproductive health, with the latter 2 comprising the largest proportion of questions.
- Early success for the FP RLAs is evident in their use to inform FP costed implementation plans, FP 2030 commitments, and the efforts of thematic working groups across multiple countries.

**Key Implications:**

- National stakeholders can leverage the process of developing FP RLAs to shape the generation and coordination of responsive research and research utilization that leads to more effective use of resources and responsive programs and policies that align with national priorities.
- Governments and funding agencies should target their investments to fund research that responds to the priorities that stakeholders have laid out in the FP RLAs, as the answers to these critical questions will improve national programs.

## INTRODUCTION

Evidence should be the foundation of well-designed family planning (FP) programs. However, the simple existence of evidence is not enough; it must be relevant, high quality, and translated into policy and practice. To advance global and country-level goals and commitments in FP, governments need to drive the development of a highly contextualized knowledge base reflective of local priorities. Translation of evidence to inform decision-making for policy and programming is an essential but often neglected step that catalyzes benefits to population health and well-being.

FP research and learning agendas (FP RLAs) can engage government and other key stakeholders to name research priorities and facilitate translation of research to practice. They build a comprehensive understanding of the FP research landscape, identify key evidence gaps, and define local priority questions to guide the production of knowledge that ushers progress toward FP goals. In addition, FP RLAs galvanize stakeholder ownership, coordination, and commitment to evidence-based policy and practice. To date, few countries have developed FP RLAs; more broadly, research is not consistently coordinated or aligned to government objectives and commitments, hindering progress.^1^

Based on our experience cocreating FP RLAs in Côte d’Ivoire, Malawi, Mozambique, Nepal, Niger, and Uganda, we argue that FP RLAs can engage stakeholders to drive the production of coordinated research that aligns with national priorities, ultimately progressing toward increasingly evidence-based programming and policy.^2^^–^^7^ In addition, country-led FP RLAs can advance global research efforts by illustrating the benefits of consistently prioritized evidence, driving investment in new research, and informing broader efforts to translate available evidence into user-friendly formats.

We argue that FP RLAs can engage stakeholders to drive the production of coordinated research that aligns with national priorities, ultimately progressing toward increasingly evidence-based programming and policy.

Historically, global health and development research has focused on topics of interest to donors, academicians, and researchers who are often based outside of the context in which the research is conducted. Increased stakeholder engagement throughout the research process can address this shortcoming. RLAs are increasingly used in the fields of global health and beyond to engage stakeholders to define knowledge gaps and prioritize questions to fill those gaps. In the field of FP, global RLAs have been recently developed to explore integrated behavior change programming,^8^ contraceptive counseling,^9^ provider behavior change programming,^10^ contraceptive-induced menstrual changes,^11^ and the hormonal intrauterine device.^12^ To date, few RLAs have focused on FP at the national level. National FP RLAs represent a unique opportunity for countries to clearly articulate what evidence is needed to help them implement national strategies, such as costed implementation plans (CIPs) for FP, and to reach national objectives and commitments. By looking across national FP RLAs, we can identify common information needs that can inform global research agendas and efforts to further package and disseminate existing evidence.

We aimed to further the evidence base in 3 technical areas originally proposed by the Research for Scalable Solutions (R4S) project and deemed critical to improving FP implementation—self-care, equity, and high impact practices (HIPs)—in 6 countries in which R4S conducts implementation science research to improve the efficiency, cost-effectiveness, and equity of FP programs.^13^^–^^15^ While these 3 technical areas were proposed for the initial inquiry and analysis processes, national stakeholders chose categories for their FP RLAs that made the most sense for their unique contexts (Table 1). For example, stakeholders in Niger and Côte d’Ivoire selected categories that align with their national CIPs for FP in addition to other areas of interest, such as task-shifting.

**TABLE 1.** Research and Learning Agenda Categories by Country

**Country**	**Categories**
Côte d’Ivoire	Demand Service delivery and access Enabling environment Coordination and monitoring High impact practices^a^ Task-shifting and self-care^a^
Malawi	Self-care^a^ Equity^a^ High impact practices^a^ Youth
Mozambique	High impact practices^a^ Self-care^a^ Equity^a^
Nepal	Approaches Global evidence (high impact practices^a^ and other evidence-based approaches) Areas for innovation (self-care^a^ and private sector) Cross-cutting areas
Niger	Demand creation Access to services Enabling environment/supervision, Monitoring and management/financing Task-shifting and self-care^a^
Uganda	Self-care^a^ Equity^a^ High impact practices^a^ Young people

a Categories originally proposed by Research for Scalable Solutions.

Though not part of the initial conceptualization of this activity, half of the countries opted to include adolescent and youth sexual and reproductive health (AYSRH) as an additional technical area for their FP RLAs. Of note, Nepal’s RLA is entirely focused on AYSRH, with self-care and equity integrated therein.

## METHODS

From mid-2020 to the end of 2021, R4S developed and coordinated a central process for cocreating FP RLAs in each of the participating countries. R4S is a 5-year FP implementation science award funded by the U.S. Agency for International Development and led by FHI 360 in partnership with Evidence for Sustainable Human Development Systems in Africa, Makerere University School of Public Health in Uganda, Population Services International, and Save the Children. A consortium partner served as the lead for each FP RLA, working with other R4S consortium partners and stakeholders; these will be referred to as the country team.

Country teams applied 3 steps to cocreate the FP RLAs: (1) a desk review of relevant policies and research and programmatic technical documents; (2) an exploration of stakeholder opinions about priority FP research topics and perspectives via key informant interviews (KIIs); and (3) consultation meeting(s) with stakeholders who reviewed the synthesized results from steps 1 and 2 and then discussed and prioritized evidence gaps and generated research and learning questions. The RLA cocreation process also involved the analysis of historical Demographic and Health Survey and Performance Monitoring for Action data, which were presented to stakeholders during consultation meetings, to identify inequities in FP service access and use. Because data availability varied considerably, and these analyses played varying roles in the production of equity-focused questions across the countries, it is not described here.

After the RLAs were completed, a central team, including staff from each partner of the R4S project, conducted analyses of the content of the 6 FP RLAs.

### Ethical Approval

Ethical clearances were obtained from each country’s national review board, as well as from the Higher Degrees, Research and Ethics Committee of Makerere University School of Public Health. FHI 360’s Office of International Research Ethics determined that the activity was not human subjects research.

### Step 1: Desk Reviews

The first step in each country involved a desk review of existing guidance documents, policies, and research evidence, including gray literature and journal articles, published between 2015 and 2020. Country team members accessed documents from a range of sources depending on the country, including sources provided by key FP stakeholders, from government and relevant FP websites, including FP2030, and through limited, targeted Internet searches. Documents were reviewed to identify any named research priorities and to ascertain evidence gaps aligned to country-specific programmatic priorities (as articulated in country-specific policy and strategy documents), as well as to the 4 technical areas of self-care, equity, and HIPs, and in some countries AYSRH (in others this area emerged through KIIs). In addition, reviewers searched documents for challenges relating to implementation. Information was extracted and organized in a spreadsheet with tabs for each technical area and an additional tab for other evidence gaps described in the literature.

In Nepal, the desk review was adapted as a landscape analysis of AYSRH-focused projects, in line with the focus on AYSRH programming. For inclusion, projects had to be implementing activities in Nepal between 2015 and 2020 that either had an FP/reproductive health (RH) outcome for young people or targeted youth programs from other sectors recommended by Nepal’s FP technical subcommittee members. The team in Nepal used Internet searches, personal communications, and requests during FP technical subcommittee meetings to identify projects that met these criteria.

### Step 2: KIIs

Country teams conducted between 12 and 25 KIIs using a structured questionnaire to identify knowledge gaps relating to key challenges in their FP programs broadly and associated with the 4 technical areas of self-care, equity, HIPs, and AYSRH. We used a convenience sample to identify interviewees; many were suggested (and approved) by the Ministry of Health and included a diverse set of FP stakeholders, including representatives of the government, implementing partners, civil society, donors, and youth-serving organizations.

The interviews were conducted by interviewers trained in qualitative interview techniques and research ethics. Interviews were conducted in English, French, or Nepali and were audio-recorded when the interviewee granted permission. Due to COVID-19, some interviews were completed virtually, while others were conducted in person. Some were completed individually, while others were in groups, depending on participant preference or other limitations. The interviews lasted approximately 1 hour. After each interview, the team reviewed the notes and recording of the interview and then wrote a summary, highlighting key themes in a standardized summary file. The country teams conducted a thematic content analysis and summarized the resulting information.

### Step 3: Consultation Meetings

Although “steps” implies a sequential nature, in many cases, steps 1 and 2 occurred concurrently and/or overlapped. In all cases, country team members and national stakeholders organized consultation meetings to review the results of the desk review and KIIs (which were synthesized in PowerPoint presentations), define and prioritize FP evidence gaps, and then formulate questions for the FP RLAs. The process for prioritizing evidence gaps and formulating questions varied by country and was led by officials from the Ministry of Health alongside the country team. As a result, the structure of each consultation meeting was unique. Each country held 1 consultation meeting, and country team members and national stakeholders determined the duration of the meetings, which ranged from 0.5 to 2 days. The number of participants at each consultation meeting ranged from 27 to 60, and participants usually represented government, international and national implementing partners, donors, and civil society, including youth-focused and youth-led organizations. Some meetings were entirely virtual, while others combined virtual and in-person settings. In some cases, we organized additional follow-up with stakeholders and/or technical committees to finalize and validate the prioritized questions, either through email communications, electronic surveys, or smaller in-person meetings. Country teams took the agreed-upon questions and drafted the FP RLA documents, which were reviewed and validated by stakeholders (either via follow-up meetings or by circulating drafts for comments) and then formally approved and signed by the appropriate national government authority.^2^^–^^7^

### Step 4: Analysis

After receiving the final FP RLAs, a subset of team members compiled all questions into a spreadsheet and categorized the questions by technical area and theme. The 4 technical areas—self-care, equity, HIPs, and AYSRH—were applied deductively to categorize the data, while themes emerged inductively from the questions. We also categorized each question as either “research”—those for which primary research would have to be conducted—or “learning”—those for which some evidence may already exist (from primary studies or pilot programs) but which may need to be synthesized, tested, or scaled up to gauge its acceptability and feasibility in a given context or those questions that can be answered outside of a formal research study. Because some questions fit within multiple categories, we selected principles to guide our classification. These included: questions that related to AYSRH and equity or AYSRH and HIPs were classified as AYSRH-focused questions; all questions related to self-care were classified as self-care.

## RESULTS/DISCUSSION

### Results Overview

A total of 349 documents were reviewed across Côte d’Ivoire, Malawi, Mozambique, Niger, and Uganda (Table 2). In Nepal, the team conducted a landscape analysis of 23 AYSRH-focused projects in lieu of a desk review. A total of 106 KIIs were conducted to surface key challenges and evidence needs from local stakeholders in each of the 6 countries. The results of the desk review and KIIs were synthesized and shared in the consultation meetings. These occurred between September 2020 and March 2021. A total of 251 attendees representing key stakeholder groups, including government, implementing partners, civil society, and donors, attended. The outcome of the process was 6 FP RLAs containing 21 to 49 questions each; together, the 6 FP RLAs comprise 190 unique questions that represent the prioritized evidence gaps for each context.

**TABLE 2.** Results of the Consultation Process

**Country**	**No. Documents**	**No. Interviews**	**Primary Consultation Date**	**Attendees**
Côte d’Ivoire	40	13	February 18, 2021	30
Malawi	75	21	October 14, 2020	57
Mozambique	68	13	March 18, 2021	27
Nepal	23	16	February 19, 2021	37
Niger	53	18	September 29–30, 2020	40
Uganda	113	25	September 24, 2020	60
Total	372	106	NA	251

Abbreviation: NA, not applicable.

We present an overview of the contents of the 6 FP RLAs organized by the 4 technical areas (Table 3), with illustrative questions and themes presented within each technical area. More information about this activity, including details as to how it was conducted and links to the 6 FP FLAs, can be accessed at https://my.visme.co/view/pvyx49me-fp-research-and-learning-agendas.^16^

**TABLE 3.** RLA Questions by Technical Area and Type of Question

	**Technical Area**					
	**Self-Care**	**Equity**	**High Impact Practices**	**Youth**	**Research Questions**	**Learning Questions **
Côte d’Ivoire	8	0	22	8	27	11
Malawi	5	3	14	9	16	15
Mozambique	5	7	10	0	9	13
Nepal^a^	0	0	0	29	14	15
Niger	6	0	9	6	9	12
Uganda	11	10	15	13	32	17
Total (% of all questions)	35 (18)	20 (22)	70 (37)	65 (34)	107 (56)	83 (44)

Abbreviation: RLA, research and learning agenda.

a All questions from Nepal’s research and learning agenda were categorized as adolescent and youth sexual and reproductive health, given this was the primary focus of the RLA. That said, self-care, high impact practices, and equity emerged as themes, and illustrative questions from Nepal are included in the tables organized by technical area (self-care, equity, high impact practices, adolescents, and youth).

Questions from Côte d’Ivoire and Niger have been translated from French. The questions that stakeholders developed are shaped by the time and context in which they were developed. For example, the presence of a global pandemic is evident in some of the questions.

### Technical Areas

#### Self-Care

The FP RLAs include a total of 35 questions related to self-care, representing 18% of all questions. While the self-care questions in the FP RLAs reflect categories included for individuals in the World Health Organization’s Classification of Self-Care Interventions, for this analysis, they were categorized based on the inductive themes of acceptability, service delivery, and supportive environment (Table 4).^17^ Questions categorized as acceptability explored subpopulations, including youth, parents, people with disabilities, and men. Within the theme of service delivery, several questions explore delivery channel or location, asking about the home, community settings, digital platforms, and pharmacies and drug shops, with some questions specifically related to self-injection of depot-medroxyprogesterone acetate (DMPA). Questions categorized under supportive environment addressed guidelines and cost effectiveness.

**TABLE 4.** Self-Care Illustrative Questions by Theme

**Theme**	**Illustrative Question**	**Country**
Acceptability	“What social norms and practices influence/affect self-care uptake in the general population and among sub-groups?”	Uganda
	“What is the level of acceptability of self-care at the community level?”	Côte d'Ivoire
	“What are strategies to mitigate negative messaging around self-care, particularly amongst adolescents and young women?”	Mozambique
	“How best can males be involved in FP self-care interventions, both as partners of eventual users and users of male-controlled self-care methods?”	Uganda
Delivery	“What do providers need to support a self-care agenda?”	Mozambique
	“What are effective practices for monitoring self-care users to minimize risk of infection?”	Niger
	“What are the enabling factors and barriers for [adolescents and youth] seeking and providing self-care services?”	Nepal
Supportive environment	“How can policies and guidelines support implementation of self-care, as defined by WHO, in Malawi?”	Malawi
	“How ready is the health system to integrate self-care?”	

Questions relating to self-care reflected the level of experience countries have with implementation. For example, in countries where there is not widespread adoption of key self-care interventions and practices and where the FP program has less self-care implementation experience—including Côte d’Ivoire, Mozambique, Nepal, and Niger—questions focused on defining needs, exploring preferences, and understanding the acceptability of self-care among various population segments. The answers to these research and learning questions can guide country programs through operationalization of self-care. In contrast, in Uganda, the only country (among the 6) that has established self-care guidelines, and Malawi, which has experience piloting and subsequently scaling up subcutaneous DMPA (DMPA-SC) across the public and private sector, questions were operational in nature, focusing on topics such as effective training, management of side effects, how to assess the impact and the readiness of the health system to scale self-care, and how to ensure that policies and stakeholders create a supportive environment for sustainable, quality self-care services.^18^

#### Equity

Equity was the focus of a total of 20 questions across the 6 FP RLAs, which were further categorized under 3 inductive themes: drivers of equity, strategies to address inequities, and fostering a supportive environment (Table 5). These themes align with steps included in the *Creating Equitable Access to High-quality Family Planning Information and Services Strategic Planning Guide*.^14^ Three equity-related questions originating in Uganda focus on drivers of equity, probing why certain types of inequities exist. The questions on drivers of inequity focus on regional, demographic, and sociocultural factors. Most (12) of the questions relate to strategies to address inequities. Some focus on a specific strategy, such as mobile outreach, community-based delivery, and private-sector provision of FP. Others present inquiries relating to strategies for addressing inequities experienced by specific populations, including those who are unmarried, sexually active young people, men/partners, and minority and marginalized groups (people living with HIV, people with disabilities, and members of minority religions). Approximately one-quarter of the equity-focused questions related to fostering a supportive environment and cover a wide range of topics, from policy to practice.

**TABLE 5.** Equity Illustrative Questions by Theme

**Theme**	**Illustrative Question**	**Country**
Drivers of equity	“Why are there regional inequities in demand satisfied by modern FP in Uganda (within and between regional variations)?”	Uganda
Strategies to address inequities	“How might the community-based service delivery models be redesigned to improve equal access and method continuation?”	Mozambique
	“What is the contribution of the private sector towards reducing the inequities in FP care?”	Uganda
Fostering a supportive environment	“How do we ensure that the definition of equity flows down from policy to practice?”	Malawi
	“How can programs be delivered more equitably among the unreached population?”	Nepal

**FIGURE 1:**
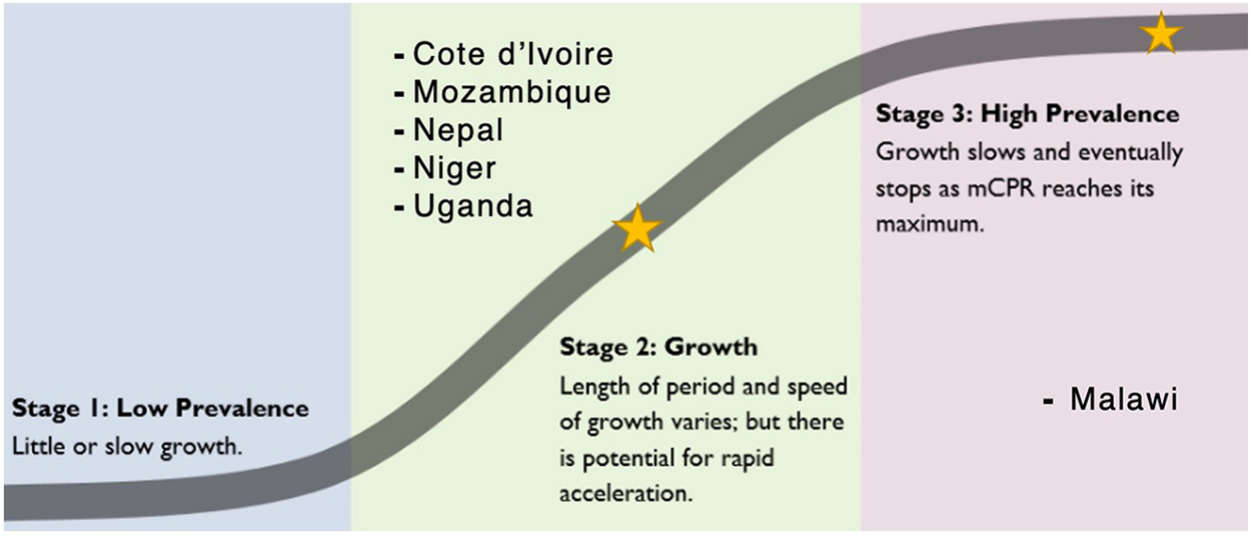
Location of Countries Preparing a Family Planning Research and Learning Agenda on the FP S-Curve^a^ Abbreviations: FP, family planning; mCPR, modern contraceptive prevalence rate. ^a^Base graph courtesy of Track20.

The FP RLAs highlighted the fact that in each of the countries, inequities continue to limit access to and use of FP and that there is much progress to be made. Questions ranged from defining equity and identifying root causes of inequity to questioning effective strategies for improving equitable access to FP. In short, all 6 countries seem to be grappling with this issue and are seeking evidence to help guide and prioritize interventions to deliver FP services equitably to their populations. That said, some countries focused equity questions on specific populations that they know are underserved. For example, in Malawi, which has the highest modern contraceptive prevalence rate and is progressing toward stage 3 of the FP S-curve (the stage where equity is prioritized),^19^ questions in the FP RLA are targeted toward young people and women living in hard-to-reach settings (e.g., those who have not already been reached by FP programs) (Figure 1).^20^^,^^21^

The FP RLAs highlighted the fact that in each of the countries, inequities continue to limit access to and use of FP and that there is much progress to be made.

Stakeholders in Mozambique and Uganda also considered adolescents and youth a key population to benefit from interventions that promote equity. Interestingly, stakeholders in both countries also included questions about how to leverage self-care to promote equity.

#### HIPs

HIPs were the largest technical area, comprising 70 questions (37%) among the 6 FP RLAs. While some country-level policy and guidance documents named specific HIPs, key informants had varying levels of familiarity with this term. As a result, evidence gaps and research questions that were broadly related to FP programming and practices were put into the HIPs technical area for the purposes of this analysis. This technical area was organized into 4 inductive themes—service delivery, social and behavior change, country leadership and coordination of partners, and costing (Table 6). The first 2 themes are the same as 2 of the 3 categories used by the HIP initiative, while the latter 2 themes align with the HIP initiative’s third category—enabling environment.

**TABLE 6.** High Impact Practices Illustrative Questions by Theme

**Theme**	**Illustrative Question**	**Country**
Service delivery	“What is the quality of FP care offered during outreaches?”	Uganda
	“What factors influence the systematic provision of immediate postpartum FP by providers?”	Côte d’Ivoire
	“How might the supply chain system be adapted to overcome stock issues and improve access to women’s preferred FP methods?”	Mozambique
	“What are the needs in terms of equipment and supplies in private health facilities to offer comprehensive FP services?”	Niger
Social and behavior change	“How effective is couples counseling in Mozambique? What are alternative effective and scalable ways to create demand for FP for male partners?”	Mozambique
	“How would men like to be engaged in FP? How do their female partners define ‘successful male engagement’?”	Malawi
Country leadership and coordination	“How can we improve alignment and coordination between FP service delivery and demand creation interventions?”	Malawi
	“What are the innovative approaches to increasing budget allocation to FP programs and reduce over-reliance on donors?”	Uganda
Costing	“What is the cost effectiveness of community-based distribution?”	Côte d’Ivoire
	“How to mobilize domestic resources to finance services and ensure the supply of contraceptive products in the absence of a financial partner?”	Niger

Of the 70 HIPs-related questions, 40% fit within the category of service delivery. These questions range from those focused on specific FP practices, such as mobile outreach services and immediate postpartum FP, to systems-level questions relating to supply chain management and private-sector provision.

Social and behavior change questions focused on levels of awareness and acceptability of services, including among certain populations. Questions related to male engagement in FP were numerous and featured in most of the FP RLAs. These were categorized as social and behavior change. It is noteworthy that questions focused on the role that social and community norms play in the acceptability and uptake of FP. For example, in Niger, the country with the lowest modern contraceptive prevalence rate among those developing FP RLAs,^22^ the FP RLA is very focused on understanding specific barriers to reaching their national objectives, including how sociocultural norms shape FP uptake, how to best generate demand for FP, and how to build a supportive infrastructure for the provision of quality FP services. This reflects Niger’s placement in the FP S-curve. Having only recently shifted from stage 1 to stage 2, they have historically emphasized increasing demand and are now beginning to address supply-side issues as well.^19^

HIPs-related questions categorized under the social and behavior change theme focused on the role that male engagement and social and community norms play in acceptability and uptake of FP.

Country leadership and coordination of partners was the second largest thematic category within HIPs. Each of the countries included at least 1 question that was focused on how to improve overall coordination. These tended to explore data sharing, documentation, and collaboration. Côte d’Ivoire, Niger, Mozambique, and Uganda each included at least 1 question related to cost and/or financing.

This exercise demonstrated that although the term “HIPs” and the associated global initiative were not always known to stakeholders across the 6 countries, they spoke of specific FP practices and were implementing different HIPs.

#### AYSRH

AYSRH emerged as an important area of focus across the RLAs, with a total of 65 questions (34% of the total). The 4 themes identified within this technical area were the role of community gatekeepers in influencing adolescent/youth decision-making, reaching youth with accurate and timely information through formal and informal channels, understanding and supporting adolescent/youth agency and decision-making, and service delivery to adolescent/youth populations (Table 7).

**TABLE 7.** Adolescents/Youth Illustrative Questions by Theme

**Theme**	**Illustrative question**	**Country**
Role of gatekeepers	“What interventions can empower parents to guide young people on matters of sexuality?”	Uganda
	“How can the behavior change interventions targeted for key influencers (parents, in-laws, gatekeepers, siblings, etc.) increase the utilization of AYSRH services?”	Nepal
	“What are effective strategies for engaging gatekeepers (parents, chiefs, religious leaders) in promoting access to FP for youth?”	Malawi
FP information	“What are the information needs of young people in terms of reproductive health and family planning?”	Niger
	“What strategies are most effective in increasing youth’s knowledge and attitudes toward FP?”	Malawi
Agency and decision-making	“What motivates young people to seek FP and RH services from the private sector as opposed to the public sector?”	Uganda
	“What are effective strategies to increase privacy & confidentiality for youth seeking FP?”	Malawi
Service delivery	“What are the reasons/causes of variations in the costs of FP services offered to young people between different health facilities?”	Côte d'Ivoire
	“How to adapt services so they are easily accessible to young people in Niger?”	Niger
	“Is there a difference in the utilization of FP/RH services in schools with adolescent-friendly information corners versus those without?”	Nepal

Abbreviations: AYSRH, adolescent and youth sexual and reproductive health; FP, family planning; RH, reproductive health.

Questions focused on the role of gatekeepers highlighted the influential positions of parents, teachers, and other local leaders in supporting (or limiting) access to FP for youth and the need for researchers to continue to engage with these groups. Questions focused on identifying effective approaches and addressing gaps in the provision of accurate and timely FP information identified a need to further understand what materials resonate best with young people and to develop strategies to battle misinformation. These questions also considered how different subsets of adolescents/youth (e.g., different age groups) may consume information and recognize the need to cater to these groups. Questions focused on better understanding and supporting adolescent/youth agency and decision-making looked to define influencing factors, as well as to identify ways to support and encourage this agency and decision-making. Finally, the FP RLA questions looking at gaps within service delivery research focused on effective and scalable approaches to ensure reliable access to affordable voluntary FP services.

Questions relating to AYSRH were frequent and varied across FP RLAs, no doubt indicating that this population group—which is the largest in size across all 6 countries—has persistent (if not growing) unmet need. Many questions addressed the role of and effective ways to engage stakeholders to support the use of FP/RH services by young people. Other questions highlighted a desire to understand what information adolescents and youth need, effectively illustrating a potential tension between what adults and young people think is appropriate in terms of AYSRH content. As researchers move to fill these evidence gaps, it will be important to strike a balance that honors stakeholders’ concerns while providing information to adolescents and youth according to the best available evidence. Under the service delivery theme, questions noted the need for a range of FP options for adolescents/youth and acknowledged the need for effective demand-creation activities in tandem with the provision of services to encourage uptake of contraception among young people.

### Research Versus Learning Questions

**FIGURE 2:**
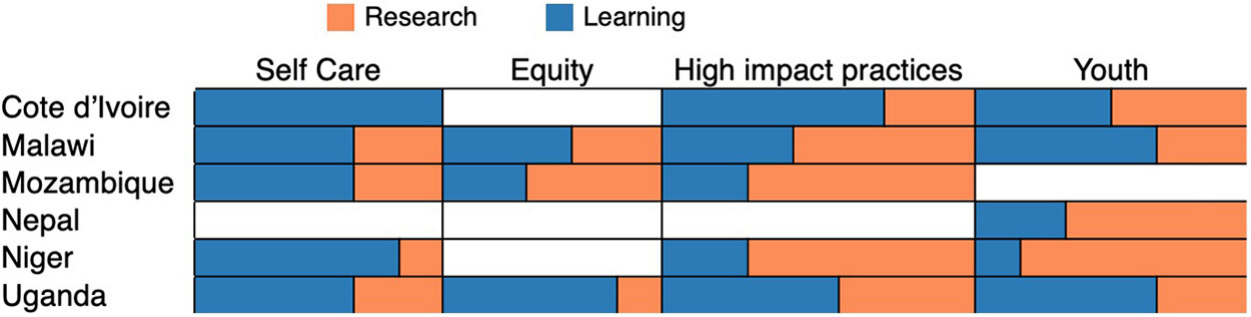
Graphic Distribution of Research and Learning Questions Across Technical Areas in the Family Planning Research and Learning Agendas Across 6 Countries

Stakeholders in the 6 countries generated questions that varied not just in technical focus but in how narrow or broadly they were worded and in terms of how the questions could be answered. As noted previously, after stakeholders in each country articulated their priority questions, 2 members of the activity team categorized them as either research or learning, the latter designation recognizing that many questions could be answered, at least partially, with existing data. Although the line between the 2 categories of questions can be blurry, Figure 2 illustrates an approximated proportion of research versus learning questions for each technical area in each country. When looking by country, Côte d’Ivoire and Uganda have more research questions in total than learning, while other countries are closer to equal proportions. When examining by technical area, HIPs and AYSRH have more learning questions overall than self-care and equity.

The high proportion of learning questions suggests a need for more translation, communication, and application of evidence and a desire for locally generated knowledge (e.g., for stakeholders to have evidence from studies implemented in their country). Translational research, research utilization, or knowledge products and dissemination events can provide support toward uptake of existing evidence when stakeholders identify this need.^23^ The AYSRH technical area illustrates this need particularly clearly. For example, the questions, “What are the best approaches to educate young people, whether in school or not, in RH/FP?” and “What type of information should we give them and through what channel?” can be answered with ample existing evidence about strategies for reaching youth with information about FP/RH through formal and informal channels. However, this evidence is not used in many countries, suggesting the need to improve translational research utilization products.^24^ In addition, in many contexts, technical working groups or subcommittees for adolescents and youth are not integrated into FP working groups; therefore, it will be important to foster effective collaboration for knowledge sharing to address AYSRH questions included in these FP RLAs.

The high proportion of learning questions suggests a need for more translation, communication, and application of evidence and a desire for locally generated knowledge.

### Significance of FP RLAs for National FP Programs and Policies

The FP RLAs were the output of a collaborative, cocreation process in each country. They were directly aligned to national commitments and objectives, allowing them to be quickly incorporated into various national processes that are shaping the future of FP in each country. The FP RLAs have been used in the preparation of CIPs in Côte d’Ivoire, Niger, and Uganda and are directly referenced in the latter 2. The FP RLA in Mozambique is similarly being discussed during the preparation of that country’s CIP. The FP RLAs have also been used in Malawi, Nepal, and Uganda as a reference during discussions to develop FP2030 Commitments. Documents have been shared and discussed in academic institutions in Nepal, as well as local, thematic working groups focused on self-care in Malawi, Niger, and Uganda, and groups on equity in Malawi, Nepal, Niger, and Uganda, providing stakeholders with a common understanding of evidence gaps and research priorities. They have also been shared with FP implementing partners to help inform project-specific monitoring, evaluation and learning plans, and related research.

Each country is exploring how to monitor the FP RLAs, with several countries proposing simple trackers to capture information about who is undertaking research that is responsive to the RLA. Monitoring trackers can be regularly shared with and updated by FP technical working groups, used to support the dissemination of relevant research results, and help avoid duplication of research across partners.

### Limitations

The original technical areas of focus for this activity—self-care, equity, and HIPs—though very important for successful national FP programs, were selected by the R4S project, which funded the development of the RLAs, as an organizing and analytical approach. Although the KII guides asked stakeholders to identify broad evidence needs and gaps, and each country selected its own organizing framework, the proposal of these specific technical areas likely meant that other topics were not included in these FP RLAs. The convenience sample used in every country for the KII may also have influenced the direction of the results, and it is very likely that some perspectives were not included, thus affecting the content of research questions and how they were prioritized. Despite these limitations, RLAs provide insights into the level and quality of information needed by FP policymakers and program planners.

The terms used throughout the process to develop the RLAs were not always familiar to stakeholders. As referenced earlier, the term HIPs to reference evidence-based FP interventions was new to many, as was the distinction between research and learning questions. Country teams worked with stakeholders in some of the countries to classify questions as either research or learning, while in other countries, this classification wasn’t used, and the categorization of the questions across countries was redone for the analysis used in this article. Finally, there are limited project-based resources to implement research that is responsive to the 6 RLAs, so the onus for funding and conducting this research will rely on continued leadership and investment at the country level.

## CONCLUSION

While FP stakeholders around the world are committed to high-quality, evidence-based FP decision-making, they may not always have the time, resources, or systematic processes available to implement locally relevant research. Policymakers and national stakeholders can cocreate FP RLAs to identify and prioritize evidence gaps and foster responsive research, thereby driving progress toward increasingly evidence-based FP programming and policy.

Despite competing priorities with the COVID-19 pandemic, stakeholders in these 6 countries were eager to engage in a process that allowed them to map their information needs to national FP priorities and to collaboratively develop research and learning questions. Country-based stakeholders report seeing value in these FP RLAs to initiate responsive research and research utilization activities to fill identified gaps and as a tool to coordinate research, ultimately leading to more prudent use of resources and higher quality, evidence-based FP programming and policy. The FP RLAs have also been shared globally with FP implementing partners and donors who gained insight into evidence gaps identified and prioritized by multiple countries—communicating an increased need for evidence translation and/or evidence generation.

Looking ahead, it may be challenging to garner commitment and support for implementation of the identified research. Governments and funding agencies should target their investments to fund research that responds to the priorities that stakeholders have detailed in the FP RLAs, as the answers to these critical questions are well-poised to improve national programs. Recent discussions about and emphasis on power-shifting and decolonization of global health and development provide further support for locally developed FP RLAs. That said, future efforts to develop national-level RLAs should allow countries to fully lead the process without imposing technical priorities. In addition, securing support to fund responsive research at the outset and liaising with researchers for a seamless transition from RLA to research would help to ensure that the RLA successfully builds up an evidence base to inform policy and practice. While it is too early to conclude if and how the priority research gaps identified in the 6 RLAs will be filled and by whom, the development of FP RLAs constitutes an important first step toward producing research that is aligned with national FP priorities, thus increasing the chances that results will be applicable and usable, and as a result more likely to improve FP outcomes.

## Notes

**Peer Reviewed**

**First published online:** August 1, 2023.
